# Integrating artificial intelligence into cancers of unknown primary diagnosis and treatment

**DOI:** 10.1016/j.isci.2026.116351

**Published:** 2026-06-17

**Authors:** Zhengzhuo Chen, Honglin Yan, Ting Xie, Lingyan Xiang, Jingping Yuan

**Affiliations:** 1 Department of Pathology, Renmin Hospital of Wuhan University, Wuchang District, Wuhan, China

**Keywords:** health sciences, medicine, medical specialty, internal medicine, oncology, natural sciences, biological sciences, systems biology, cancer systems biology, applied sciences, computer science, computing methodology, artificial intelligence

## Abstract

Cancers of unknown primary (CUP) refer to a highly heterogeneous group of metastatic tumors whose primary site remains undetectable despite comprehensive conventional evaluations. Characterized by obscure primary origins and extremely poor prognosis, the pathogenesis of CUP remains incompletely elucidated. Empirical chemotherapy, the traditional mainstay of treatment, yields limited efficacy, while emerging therapeutic strategies lack sufficient evidence from randomized controlled trials. Consequently, CUP continues to pose a formidable challenge in clinical practice. However, advances in artificial intelligence (AI), particularly in deep learning, have enabled reliable performance in oncology-related tasks, including tumor diagnosis, treatment response prediction, and prognostic assessment. In CUP research, AI is predominantly applied to develop diagnostic models for predicting tumor tissue of origin (TOO) or molecular subtypes, with a small number of studies focusing on the prediction of treatment response and survival. Current AI models that integrate multi-modal data (e.g., molecular data, medical imaging) leverage their advantages in high-throughput data processing and in-depth feature mining to overcome the limitations of traditional CUP diagnosis and treatment, providing new avenues to better understand this complex disease. Given the substantial progress of AI in CUP, this review systematically summarizes the current research status and latest breakthroughs of AI in CUP from three perspectives: AI-based diagnosis using molecular data, AI-based diagnosis using medical imaging, and AI-assisted prediction of treatment response and prognosis. The aim is to promote precise diagnosis and treatment of CUP and improve patient outcomes.

## Graphical abstract

**Figure:**
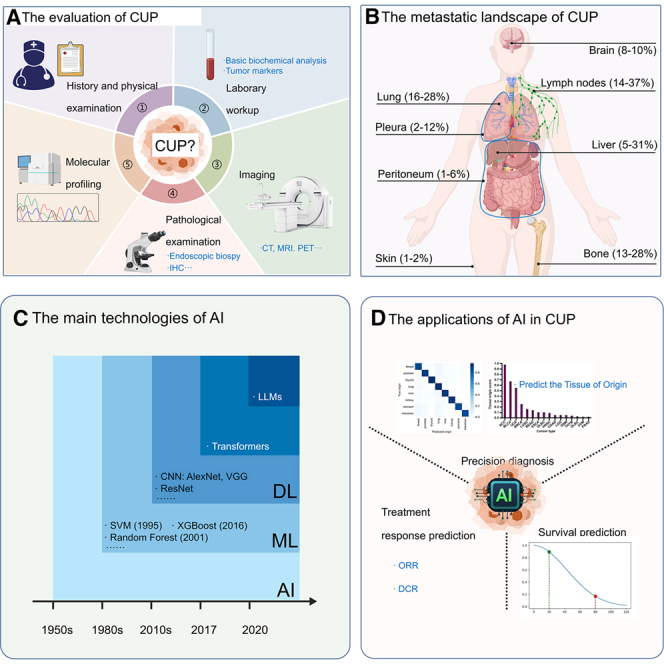

## Introduction

**Figure 1:**
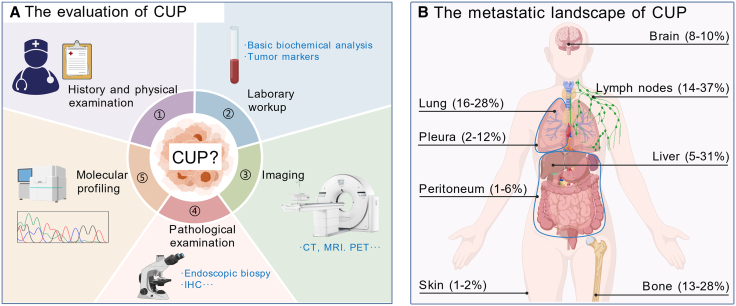
Overview of CUP (A) Evaluation of CUP. The current evaluation process for CUP includes detailed medical history and physical examination, laboratory tests (e.g., routine biochemical analysis, tumor marker detection), imaging studies, pathological examination (e.g., endoscopic biopsy, IHC testing), and molecular profiling (e.g., genomics, transcriptomics, epigenomics). (B) Metastatic landscape of CUP. Annotations indicate the major metastatic sites of CUP and their statistical proportions.

Cancers of unknown primary (CUP) are defined as highly heterogeneous metastatic tumors, where the primary site remains undetectable after comprehensive evaluations, including detailed medical history taking, physical examination, tumor marker testing, imaging studies, pathological examination, and immunohistochemistry (IHC) (Figure 1A).^1^^,^^2^^,^^3^^,^^4^ CUP can present as single or widespread metastases involving various anatomical sites, such as lymph nodes (14%–37%), liver (5%–31%), lungs (16%–28%), brain (8%–10%), bones (13%–28%), and serous cavities (pleura, 2%–12%; peritoneum, 1%–6%) (Figure 1B).^5^^,^^6^ The underlying mechanisms of CUP remain poorly understood. Existing theories propose two main hypotheses: (1) the primary tumor may regress due to host immune suppression, becoming undetectable by current diagnostic techniques; (2) premalignant or malignant stem cells may migrate to distant sites and form metastatic lesions without establishing a primary tumor at the original location.^1^^,^^7^

The incidence of CUP varies slightly across regions, with an overall rate of 6–16 per 100,000 individuals, ranking it as the eighth most common cancer.^8^^,^^9^^,^^10^^,^^11^^,^^12^^,^^13^ Notably, advancements in diagnostic technologies have led to a declining incidence, with CUP now accounting for 1%–2% of all cancers, down from the historical 3%–5%.^14^ Nevertheless, CUP remains the fourth leading cause of cancer-related deaths and poses a grave threat to patient survival.^15^^,^^16^ In current clinical practice, the diagnosis of metastatic tumors and CUP relies heavily on comprehensive imaging and pathological examinations. However, imaging modalities have limited ability to localize small lesions or widespread metastases.^17^^,^^18^^,^^19^ Pathological examination demonstrates suboptimal diagnostic accuracy especially for poorly differentiated or undifferentiated tumors,^20^ and IHC often consumes limited tumor tissue, thereby limiting subsequent molecular testing.^21^ In terms of treatment, most CUP patients still receive platinum-based empirical chemotherapy with limited clinical benefit.^22^ With the emergence of multiple high-quality clinical trials, novel therapeutic strategies—including tumor tissue of origin (TOO)-directed therapy (predicting primary sites based on IHC^23^ or molecular profiling^24^), molecularly guided targeted therapy,^25^^,^^26^ and immunotherapy^27^^,^^28^—have demonstrated significant clinical value. Additionally, due to the absence of a definitive diagnosis, CUP patients often undergo a prolonged and arduous diagnostic journey, leading to significant psychological distress.^29^^,^^30^ Thus, there is an urgent need for more precise and efficient diagnostic and therapeutic strategies to address the current bottlenecks in CUP management.

Advances in AI, particularly in deep learning (DL), have profoundly impacted medical research and clinical practice.^31^ Compared to traditional techniques, AI excels in processing multimodal high-throughput data, mining latent features that are inaccessible to the human eye or conventional algorithms, generating high-order features from primary data, and constructing predictive models by mapping these features to clinical outcomes. These strengths enable AI to achieve robust performance in tumor detection, assisted diagnosis, treatment response prediction, and prognostic assessment.^31^ In CUP research, most AI studies focus on developing diagnostic models to predict TOO, histological subtypes, or molecular subtypes, whereas only a small proportion of studies investigate treatment response and survival prediction.^32^ The prevailing research paradigm for CUP diagnostic modeling is to train, internally validate, and externally validate models using large-scale datasets of tumors with confirmed diagnoses, and then apply the trained models to CUP cohorts. Notably, the reference labels for CUP cohorts are usually determined by expert panels through comprehensive analysis of detailed clinical and investigative findings; yet these labels carry inherent uncertainty due to the intrinsic nature of CUP. Current AI diagnostic models for CUP are mainly built on molecular data and medical imaging data, and often need to classify dozens of tumor types, which poses substantial challenges to their performance.

Given the remarkable progress and vast potential of AI in CUP, this review systematically summarizes the current status and latest advancements of AI applications in CUP from three aspects: “AI-based diagnosis using molecular data, AI-based diagnosis using medical imaging, and AI-assisted prediction of CUP treatment response and prognosis,” with the goal of promoting precision management.

## Overview of AI

**Figure 2:**
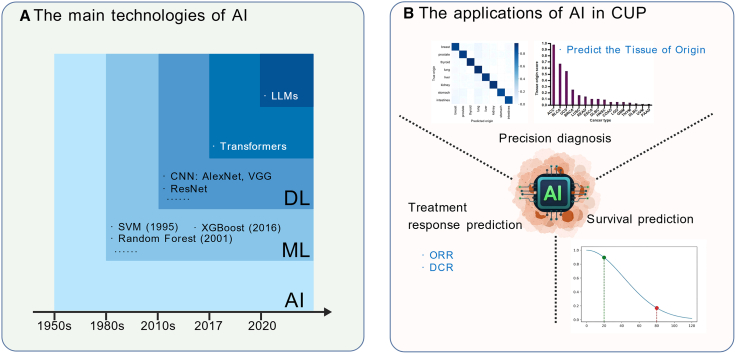
Overview of AI (A) Core technologies of AI. The horizontal axis represents the years when various technologies emerged. (B) Applications of AI in CUP. AI is mainly used for precise diagnosis, treatment response prediction, and survival prognosis prediction in CUP.

Artificial intelligence (AI) encompasses a broad range of technological systems designed to simulate human intelligence through computer algorithms. Its core technologies include machine learning (ML) and DL, where ML is a key subfield of AI, and DL is a specialized branch of ML (Figure 2A).^33^

Classical ML algorithms automatically learn patterns from data via statistical models, typically requiring manual feature engineering to perform classification, prediction, or other tasks. ML is particularly effective for problems with moderate data sizes and low-dimensional features, with representative algorithms including random forest, support vector machine (SVM), and XGBoost.^34^

DL is characterized by deep neural network (DNN) architectures,^35^ which are typically composed of an input layer, multiple hidden layers, and an output layer. Unlike classical ML, DL automatically extracts complex, abstract features from large-scale high-dimensional data through multi-layered structures, enabling more accurate task execution without relying on manual feature design. Common DNN architectures include: (1) convolutional neural networks (CNNs)—these networks employ convolutional and pooling layers to extract and condense features, with classical models such as AlexNet and VGG. (2) Residual neural networks (ResNets)—by introducing residual connections, ResNets mitigate the vanishing gradient problem in very deep networks and have become one of the most widely used backbone architectures. (3) Transformer architectures—the transformer, built on a self-attention mechanism, captures global dependencies within sequences without relying on convolutional operations for local feature extraction.

In recent years, large language models (LLMs) based on transformer architectures and pre-trained on massive text data have significantly advanced AI capabilities,^36^ which not only handle multimodal data (e.g., text and images) but also perform a diverse range of tasks, including classification, regression, and natural language interaction.

## AI-based diagnosis of CUP using molecular data

**Figure 3:**
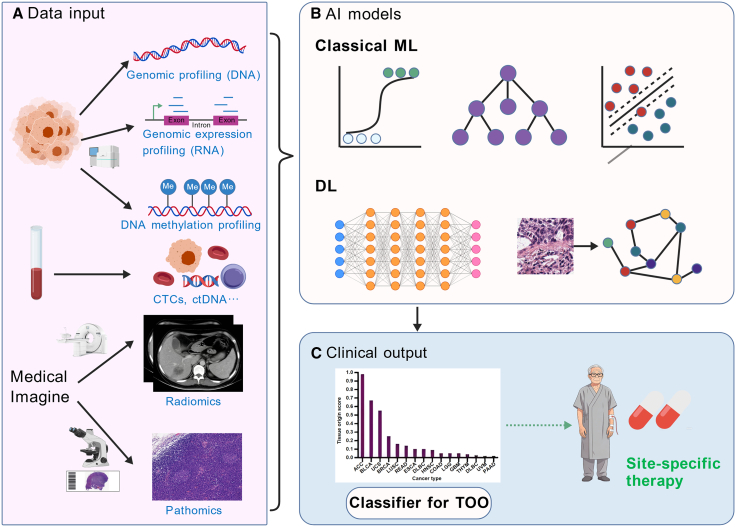
AI-based diagnosis in CUP using multimodal data type (A) Data input. It includes molecular data (e.g., genomics, transcriptomics, epigenomics) and medical imaging data (e.g., radiological images, pathological images). (B) AI models. Diagnostic models are developed using ML and deep learning approaches. (C) Clinical output. The final output provides predictions for the tissue of origin or molecular subtypes, which can inform clinical management.

CUP is pathologically confirmed metastatic tumor, and the primary aim in developing diagnostic models for CUP is to predict the tissue of origin. The tissue-specific patterns of molecular alterations in tumor provide a fundamental theoretical basis for developing TOO prediction models from molecular data. Currently, AI models built on various molecular datasets—including genomics, transcriptomics, epigenomics (Figure 3A)—have demonstrated remarkable performance in overcoming traditional diagnostic limitations.^37^

### AI-based diagnosis using genomic data

DNA molecules are highly stable, rendering genomic data an ideal resource for developing TOO prediction models. Current genomic sequencing technologies include whole genome sequencing (WGS), whole exome sequencing (WES), and targeted panel sequencing. WGS provides a comprehensive profile of the genome, enabling the detection of all genetic variants, including viral integrations and chromosomal structural alterations. In contrast, WES and targeted panel sequencing employ specific primers to capture predefined genomic regions, a strategy that improves efficiency but may omit substantial portions of the non-targeted genome (Table 1).

**Table 1:** Summary of AI models for CUP diagnosis based on molecular data

Reference	Multimodel data type	AI type	Training *n*	Classes	Test performance	CUP cohort *n* & performance
Nguyen et al.^38^	genomic WGS	ML	6,756	35	ACC≈90%	141, ACC = 58%
Schipper et al.^39^	genomic WGS	ML	4,509	29	ACC = 84%	72, ACC = 68%
Sanjaya et al.^40^	genomic mutation	DL	9,939	29	ACC = 64%–89%	not validated
Zhang et al.^41^	copy number variations	ML	4,566	10	ACC = 89.1%	not validated
Soh et al.^42^	somatic mutations and copy number alterations	ML	6,640	28	ACC = 77.7%	not validated
Moon et al.^43^	genomic targeted panel	ML	36,445	22	F1-score = 94.2%	971, 41.2% (high-confidence)
Penson et al.^44^	genomic targeted panel	ML	7,791	22	ACC = 74.1%	not validated
Darmofal et al.^45^	genomic targeted panel	DL	39,787	38	ACC = 92.7%	not validated
Jiao et al.^46^	genomic mutation	DL	2,606	24	ACC = 91%	not validated
Salvadores et al.^47^	genomic passenger mutations	ML	2,267	18	ACC = 92%	not validated
Zhao et al.^50^	transcriptome	ML	18,217	32	ACC = 96.7%	not validated
He et al.^54^	transcriptome	ML	9,911	32	ACC = 91.1%	not validated
Divate et al.^55^	transcriptome	DL	14,237	37	ACC>97%	not validated
Shen et al.^58^	transcriptome	ML	10,553	24	ACC = 91%–94%	not validated
Raghu et al.^59^	microRNA	DL	9,648	18	ACC = 97%	not validated
Xie et al.^65^	DNA methylation	DL	12,039	32	F1-score = 89%	not validated
Ning et al.^75^	DNA methylation	ML	8,296	26	ACC = 88%	not validated
Abraham et al.^77^	genome + transcriptome	ML	77,044	21	ACC = 94%	not validated
Liu et al.^79^	circulating methylated cfDNA	ML	6,689	50	ACC = 93%	not validated
Conway et al.^81^	circulating methylated cfDNA	ML	9,017	29	ACC = 96.8%	41, 88.5% clinical consistency

Nguyen et al.^38^ developed a ML model, named CUPL, for TOO prediction. This model employed a random forest algorithm to select 511 features from WGS data of 6,756 tumors. And it achieved approximately 90% accuracy and 90% recall in classifying 35 cancer types/subtypes, with 58% accuracy in a cohort of 141 CUP patients. Similarly, Schipper et al.^39^ developed a ML classifier, CUPPA, for TOO prediction using tumor WGS data. The model demonstrated 84% accuracy in external validation (29 tumor types) and 68% accuracy in a 72-patient CUP cohort.

In contrast to conventional ML algorithms that use simplified feature extraction, Sanjaya et al.^40^ developed a TOO classifier using a DL architecture termed mutation-attention (MuAt). This framework integrates detailed information of genetic mutations—encompassing variant types and genomic location—rather than simplified mutation aggregate counts. This model achieved 89% accuracy among 29 tumor types. Additionally, several studies have shown that incorporating copy number variation (CNV) features can significantly improve model performance.^41^^,^^42^

In clinical practice, WGS and WES are limited by high costs and low efficiency, while targeted panel sequencing is more widely used due to its cost-effectiveness and practicality. Thus, TOO prediction models based on targeted panel sequencing data are more conducive to clinical translation. Moon et al.^43^ developed OncoNPC, a ML classifier trained on data from 36,445 tumors (300–500 genes), which achieved a weighted F1-score of 0.942 in classifying 22 tumor types. Notably, when applied to a cohort of 971 CUP patients, OncoNPC provided high-confidence predictions (>95%) for 41.2% of cases. In another study, Penson et al.^44^ developed a random forest classifier for TOO prediction using targeted panel sequencing data (468 genes) from 7,791 tumors, reporting 74.1% accuracy in external validation. Darmofal et al.^45^ developed a DL model based on targeted panel sequencing data (341 genes), integrating multiple genomic features including mutations and insertions, focal amplifications and deletions, broad copy number alterations, structural rearrangements and fusions, mutational signatures, tumor mutational burden (TMB), and microsatellite instability (MSI) scores. This model achieved 92.7% high-confidence prediction accuracy across 38 tumor types, representing improved classification breadth and accuracy compared to previous studies, highlighting the value of integrating multi-dimensional genetic features for model performance enhancement.

Another study^46^ explored the impact of passenger mutations (abundant non-functional alterations) and driver mutations (functional oncogenic mutations) on TOO prediction model performance. A DL model developed using passenger mutations achieved 91% accuracy in predicting 24 tumor types. Surprisingly, the addition of driver mutation features reduced model performance, indicating that passenger mutations provide more TOO-related information than driver mutations—challenging the traditional notion that driver mutations are more diagnostically valuable.^47^

### AI-based diagnosis of CUP using transcriptomic data

Current transcriptomic profiling platforms mainly include RNA sequencing (RNA-seq) and microarray technologies. RNA-seq enables quantification of absolute gene expression levels and identification of structural variations (e.g., gene fusions, alternative splicing) but is relatively costly. Microarray technology uses specific probes to improve sequencing efficiency and reduce costs but only provides relative gene expression levels.

Numerous ML models for TOO prediction have been developed based on RNA-seq data,^48^^,^^49^^,^^50^^,^^51^^,^^52^^,^^53^^,^^54^ using algorithms such as logistic regression, random forest, and SVM, all achieving high performance. For instance, a model developed by He et al.^54^ reported 91.1% top-1 accuracy and 97.5% top-3 accuracy in an independent validation cohort. Zhao et al.^50^ developed CUP-AI-Dx, which achieved 96.7% accuracy across 32 tumor types and could further identify 11 tumor molecular subtypes, providing valuable guidance for clinical decision-making. With the application of DL, model accuracy has been further improved. For instance, Mayur et al.^55^ trained a DL neural network model using pan-cancer gene expression data, achieving 97% accuracy in classifying 37 tumor types.

However, due to differences in data formats between microarray and RNA-seq platforms, models developed on one platform are often incompatible with the other. Research teams led by Frans^56^ and Curr^57^ addressed this issue by integrating data from both platforms using gene set enrichment scores, achieving 0.85 and 0.86 accuracy in external validation, respectively. Additionally, Shen et al.^58^ developed a majority voting algorithm based on gene expression ranks to enable cross-platform compatibility. By selecting 538 feature genes based on expression ranks for TOO prediction, their model attained 91%–94% accuracy in multiple external validation cohorts. Notably, it has been deployed as a web-based tool to facilitate easy access and utilization.

Beyond protein-coding RNA, several studies have developed TOO prediction models using microRNAs (miRNAs), a class of non-coding RNAs that regulate gene expression. miRNAs exhibit high stability and resistance to degradation compared to other RNA molecules, making them ideal for TOO classification. Ananya et al.^59^ developed a DL model based on miRNA data to predict the origin of 18 cancer types, achieving over 90% accuracy in 15 types, demonstrating the great potential of miRNAs for TOO prediction.

### AI-based diagnosis using epigenetic data

In mammalian cells, DNA methylation serves as a pivotal epigenetic mechanism governing tissue-specific gene expression, characterized by high stability and heritability. Current DNA methylation profiling platforms primarily comprise the 450 K array and 850 K BeadChips, which differ significantly in probe design and coverage. Several research teams have developed TOO classifiers using ML^60^^,^^61^^,^^62^^,^^63^ or DL^64^ based on public methylation data, achieving accuracy ranging from 0.85 to 0.96. Xie et al.^65^ integrated methylation data from both 450 and 850 K platforms to construct a transformer model that incorporated pathway crosstalk information. This model attained an average F1-score of 89% in classifying 32 cancer types, effectively addressing cross-platform data compatibility issues and enhancing clinical applicability.

As CUP is a metastatic disease, the anatomical location of metastases can provide critical clues regarding the primary tumor origin. Thus, several studies have developed site-specific classifiers for specific anatomical regions, such as head and neck metastatic squamous cell carcinoma,^66^^,^^67^ sinonasal tumors,^68^ central nervous system tumors,^69^ and sarcomas.^70^^,^^71^ These site-specific models require smaller training datasets and lower training costs while offering enhanced discriminative power. For example, distinguishing between liver metastases from pancreatic ductal carcinoma and intrahepatic cholangiocarcinoma is clinically challenging; nevertheless, Teodor et al.^72^ developed a diagnostic model that achieved 94.28% accuracy for this specific task. Similarly, Philipp et al.^73^ developed a ML model based on DNA methylation data to discriminate pulmonary enteric adenocarcinoma from metastatic colorectal cancer.

Conventional modeling approaches typically rely on the extraction of high-dimensional features, which may lead to model overfitting. Constructing efficient diagnostic models with low-dimensional features remains a significant methodological challenge.^74^ Ning et al.^75^ developed a pan-cancer diagnostic model using only 30 methylation sites, selected through optimal overlap frequency and feature ranking algorithms, achieving an average accuracy of 0.88 across 26 cancer types. Similarly, Adam et al.^76^ constructed a classifier based on organ-specific quantitative trait loci (QTLs), achieving 93.12% accuracy in the classification of six tumor types.

### AI-based diagnosis using multi-omics data

Multi-omics data integration combines molecular data—including genomics, transcriptomics, epigenomics—to construct comprehensive tumor molecular profiles, thereby overcoming the limitations of single-omics data. Jim et al.^77^ integrated genomic and transcriptomic data to develop a ML model for TOO prediction using MI GPSai (genomic prevalence score), achieving 94% top-1 accuracy in a test cohort of 19,555 patients and demonstrating superior performance compared to single-omics models. He et al.^78^ used random forest algorithms to integrate 6 genetic mutations and 74 gene expression features, constructing a multi-omics model that attained 89% accuracy.

### AI-based diagnosis using circulating molecular data

Circulating molecular data refers to molecular profiles derived from liquid biopsy specimens. When tumor cells die or undergo apoptosis, they release nucleic acid fragments into the bloodstream; while most are degraded, a small fraction remains stable and detectable, including circulating cell-free DNA (cfDNA) and miRNAs. Compared to invasive tissue biopsy, which carries inherent risks of bleeding, injury, and infection, liquid biopsy offers non-invasive advantages and enables early screening, making it an ideal choice for patients with inaccessible tissue samples.

A major challenge in developing tumor diagnostic models based on circulating molecular data are the high heterogeneity of tumor-derived circulating molecules in plasma and interference from background molecules (e.g., leukocyte-derived nucleic acids). Liu et al.^79^ performed targeted methylation sequencing of plasma ctDNA in a prospective study of ∼6,700 patients, developing a ML model for cancer detection and TOO classification with 93% accuracy. Zhou et al.^80^ developed a semi-reference-free deconvolution (SRFD) algorithm to automatically learn reference databases from cfDNA methylation features, eliminating the need for manual alignment with tissue-derived reference databases. Based on this, they constructed a Bayesian diagnostic model (SRFD-Bayes) combining tumor fraction and ML classification, achieving 86.1% sensitivity in early cancer detection and 76.9% accuracy in TOO prediction across 6 tumor types. Unfortunately, limited by the difficulty of CUP cohort recruitment, these two studies have not been validated in real CUP cohorts.

To address the high variability of ctDNA content in cfDNA, Alicia-Marie et al.^81^ mixed public tissue DNA methylation data from The Cancer Genome Atlas (TCGA) with self-generated cfDNA methylation data to build a ML model (CUPiD), achieving 96.8% accuracy in external validation for TOO prediction. Notably, the model was evaluated in a CUP cohort of 41 patients, and 88.5% of its predictions were clinically consistent with subsequent or suspected primary tumor diagnoses where available.

## AI-based diagnosis of CUP using medical imaging

AI models based on molecular data have shown excellent performance in TOO prediction and demonstrated potential in CUP cohorts. However, comprehensive molecular profiling is not routinely performed into the clinical workflow for metastatic tumor patients, especially in resource-limited settings. In contrast, radiological imaging and histopathological examinations are more accessible, making AI models based on medical imaging a valuable complement (Table 2).

**Table 2:** Summary of AI models for CUP based on medical imaging

Reference	Multimodel data type	AI type	Training n	Classes	Test performance	CUP cohort n & Performance
Xin et al.^90^	liver CT	DL	3,105	6	ACC = 85.2%	not validated
Lyu et al.^92^	brain MRI	DL	1,582	5	ACC = 81%–88%	not validated
Liu et al.^95^	spine MRI	DL	295	5	AUC = 77%	not validated
Lu et al.^96^	histopathology WSI	DL	6,499	18	ACC = 80%	317, top-1 = 61%
Zhu et al.^97^	bone biopsy pathology WSI	DL	1,473	8	ACC = 91%	175, 73.14% consistent with IHC
Zheng et al.^98^	cervical lymph node pathology WSI	DL	1,036	5	AUC = 98%	not validated
Tian et al.^99^	cytological smear WSI of serous cavity effusion	DL	57,220	5	ACC = 82.6%	391, top-1 = 78.8%

### AI-based diagnosis using radiological images

AI models based on radiological images (computed tomography [CT], magnetic resonance imaging [MRI], ultrasound) have exhibited excellent performance in tumor segmentation and treatment response prediction. AI can extract subtle features invisible to the human eye and correlate them with classification tasks. However, most existing studies classify metastatic tumors as a single category without predicting their primary origin,^82^^,^^83^^,^^84^^,^^85^^,^^86^^,^^87^^,^^88^^,^^89^ likely due to limitations in the volume of metastatic tumor imaging data and model classification capabilities. Currently, AI-based TOO prediction for metastatic tumors using radiological images mainly focuses on solid organ tumors (liver,^90^ brain,^91^^,^^92^^,^^93^ lung^94^) and bone metastases.^95^

For example, Xin et al.^90^ developed a ResNet-based architecture to predict the origin of liver metastases from multi-phase contrast-enhanced CT images, achieving an average accuracy of 85.2% across six primary tumor types. Lyu et al.^92^ developed a transformer-based model to predict the origin of brain metastases from MRI images, achieving 81.3%–88.2% accuracy for five tumor types. For non-visceral metastases, Liu et al.^95^ developed a ResNet-50 model based on spinal MRI images to predict the origin of 5 metastatic tumor types, achieving an area under the curve (AUC) of 0.77. AI assistance is shown to improve the diagnostic accuracy of radiologists.^89^ Nevertheless, current radiological AI models for TOO prediction lack validation in CUP cohorts. Furthermore, there is an absence of radiological AI models for metastases in lymph nodes or other occult anatomical sites.

### AI-based diagnosis using pathological images

Pathological diagnosis is widely regarded as the “gold standard” for tumor diagnosis, as pathological images contain rich microstructural information, including tissue architecture and cellular morphology. Recent advances in scanner technology have enabled the digitization of pathological sections into whole slide images (WSIs), providing a data foundation for AI analysis. Developing AI models for pathological images typically requires preprocessing steps such as WSI cropping and color normalization. DL applied to pathological images can identify subtle features invisible to the human eye, enabling the development of models for various downstream tasks based on image biomarkers.

A landmark breakthrough in DL-based CUP diagnosis using histopathological images was the development of the TOAD model by Lu et al.^96^ in 2021. TOAD can identify the origin of 18 tumor types from histopathological images, achieving 0.80 top-1 accuracy and 0.93 top-3 accuracy in external validation, demonstrating high clinical applicability and generalization. In a test cohort of 317 CUP patients, TOAD achieved 0.61 top-1 accuracy and 0.82 top-3 accuracy, comparable to the diagnostic performance of pathologists. Furthermore, TOAD-assisted diagnosis can narrow down the range of potential primary sites, reducing the need for IHC and preserving limited paraffin-embedded tissue samples.

In clinical pathological workflows, pathologists often know the biopsy site, which provides crucial clues to the primary tumor origin due to anatomical drainage patterns. Consequently, developing site-specific models for pan-cancer metastasis diagnosis can achieve high performance with moderate training data volumes. For example, Zhu et al.^97^ developed a DL model to predict the origin of bone metastatic tumors using histopathological images from 1,473 patients. This model achieved 91.35% top-1 accuracy and 97.75% top-3 accuracy in internal validation, and 97.44% top-3 accuracy in external validation. When applied to a cohort of 175 CUP patients, the model’s predictions demonstrated high consistency (73.14%) with TOO suggested by IHC markers. Similarly, Zheng et al.^98^ developed a DL model to predict the origin of cervical lymph node metastatic cancer using pathological images, achieving an impressive AUC of 98.02% for five tumor types.

A significant proportion of newly diagnosed CUP patients present with malignant pleural or peritoneal effusions. For patients unable to tolerate invasive surgical procedures, obtaining sufficient tissue for histopathological examination is often challenging; consequently, cytological examination of effusions plays a pivotal role in CUP diagnosis. Tian et al.^99^ developed a DL model (TORCH) to predict TOO from pleural and peritoneal effusion cytology images, achieving 82.6% top-1 accuracy across five tumor types. In a comparative study with four pathologists, TORCH achieved 78.8% top-1 accuracy, significantly outperforming the pathologists, demonstrating strong clinical applicability. However, cytological examination has limitations, as smears may not fully represent the entire liquid sample. A technique called “cell block” is increasingly used for pathological examination of liquid samples, involving centrifugation of liquid specimens to concentrate tumor cells, followed by paraffin embedding, hematoxylin and eosin (HE) staining, and IHC. Cell block technology improves tumor cell detection rates and diagnostic accuracy compared to conventional cytological smears.^100^ Zhang et al.^101^ developed a DL model based on HE-stained cell block images for adenocarcinoma screening in serous cavity effusions. However, this study did not further explore TOO prediction, possibly due to limited sample size.

## AI-assisted prediction of treatment response and prognosis in CUP

Extensive evidence confirms that AI serves as a transformative paradigm in precision oncology, capable of accurately forecasting therapeutic efficacy and patient survival outcomes, thereby significantly advancing the field of precision tumor treatment.^102^ However, studies on efficacy and prognosis prediction using AI methods in CUP patients are relatively scarce, which may be closely related to the lack of treatment follow-up data.

OncoNPC, a tumor origin prediction model developed based on targeted panel genomic data, showed in a retrospective survival analysis that CUP patients who received treatment consistent with OncoNPC predictions had significantly longer survival time than those who received treatment inconsistent with the predictions.^43^ Another tumor origin prediction model TORCH, developed based on pathological images of pleural and ascitic cytology smears, was applied to a cohort of 391 CUP patients receiving empirical treatment. The results showed that patients whose treatment regimens were consistent with the tissue origin predicted by the TORCH model had significantly better efficacy and survival than those with inconsistent regimens.^99^ The above studies suggest that AI intelligent models can effectively assist in the efficacy prediction of CUP patients and have potential value in guiding clinical treatment.

The overall prognosis of CUP patients is poor.^103^^,^^104^^,^^105^^,^^106^ Current clinical consensus divides patients into “favorable subgroups” with relatively good prognosis (accounting for about 20%, such as isolated lymph node metastasis, adenocarcinoma with characteristics of prostate cancer, breast cancer, colon cancer, etc.) and “unfavorable subgroups” with extremely poor prognosis (accounting for about 80%, mostly with extensive metastasis).^107^ In addition, existing studies have explored a variety of biomarkers with prognostic predictive value, such as skeletal muscle density,^108^ systemic inflammatory markers,^109^^,^^110^ etc. In the past, some studies used traditional statistical methods such as Cox regression analysis for prognosis prediction of CUP patients,^111^^,^^112^ but these methods are not AI technologies. It is worth noting that Cui et al. constructed a prognostic prediction model using ML algorithms to predict the 3-month survival rate of CUP patients with bone metastasis, and the accuracy of the model in external testing reached 74.5%.^113^ In the future, there is an urgent need to develop efficacy and prognosis prediction models for CUP based on multimodal data using DL methods to provide guidance for clinical treatment and further improve the prognosis of CUP patients.

## Limitations and future perspectives

### Limitations

#### Insufficient biological interpretability and clinical trust barriers of AI models

Current AI models in the CUP field suffer from severe insufficient biological interpretability. Most AI models based on medical imaging improve interpretability using attention heatmaps, yet fail to link morphological features to underlying tumor biological mechanisms. However, interpretability analysis is nearly absent in current molecular data-based AI models. In fact, features extracted by AI from molecular data may help identify novel markers for TOO. The inherent “black box” nature of AI significantly reduces clinicians’ trust in model predictions. Therefore, there is an urgent imperative to develop biologically interpretable AI models and subject them to adequate prospective clinical validation.

#### Class imbalance and diagnostic bottlenecks of rare subtypes

Existing AI models are mostly trained on public pan-cancer datasets such as the TCGA cohort, which are dominated by common cancers with extremely limited samples of rare tumor subtypes, resulting in severe class imbalance. CUP presents distinct clinical characteristics, and its patient population harbors a far higher proportion of rare tumor biological phenotypes than conventional cancer populations. This leads to a substantial mismatch between training datasets and real-world CUP clinical scenarios. Such discrepancy renders current models incapable of effectively identifying rare subtypes that account for a considerable proportion of CUP cases, accompanied by poor diagnostic accuracy, which has become a critical bottleneck restricting precise CUP diagnosis.

Moreover, numerous studies have demonstrated a marked performance decline when existing models are applied to CUP cohorts. This phenomenon can be attributed to in-depth biological mechanisms, including epigenetic reprogramming during tumor metastasis, clonal evolution, immune-mediated primary tumor regression, and the failure of current datasets to reflect the unique biological characteristics of CUP. Collectively, these factors impair the practical diagnostic performance of AI models.

#### Restricted generalization performance caused by multiple heterogeneities

First, batch effects inherent in public databases undermine model training stability and reduce its adaptability across diverse datasets and clinical settings.

Second, technical heterogeneity exists across medical institutions, including differences in whole-slide imaging scanners, tissue staining protocols, tissue fixation time, and sample processing procedures. Such technical variations directly affect feature extraction and recognition accuracy of pathological AI models, thereby weakening their cross-center generalization ability.

Third, there is heterogeneity in diagnostic criteria for CUP cohorts. Although most studies follow the European Society for Medical Oncology (ESMO) clinical guidelines, subtle discrepancies still exist in diagnostic standards. Furthermore, customized diagnostic criteria adopted by several research centers are not fully clarified. This not only hinders the horizontal comparison of model performance across studies, but also increases the complexity of model validation and impairs the reliability and generalizability of validation outcomes.

#### Fairness and accessibility

Core technologies for CUP diagnosis, including molecular sequencing and pathological whole-slide scanning, are constrained by uneven medical resource distribution and difficult to popularize in low- and middle-income regions worldwide. Notably, these resource-limited areas are faced with insufficient epidemiological data and inadequate research on CUP. Consequently, existing research findings cannot meet local clinical demands, making equitable and universal access to standardized CUP diagnosis difficult to achieve.

### Future perspectives

Despite the prevailing challenges confronting AI applications in the CUP field, its potential to surmount traditional diagnostic and therapeutic bottlenecks and to catalyze precision medicine remains unequivocal. Given that most existing models are constructed using single-modality data, next-generation frameworks leveraging multi-omics integration are poised to substantially enhance diagnostic performance. Furthermore, propelled by the rapid evolution of LLMs, we anticipate the development of CUP-specific foundational models. These advanced systems are envisioned to seamlessly integrate diagnosis, prognostic prediction, and patient management into a unified clinical workflow, thereby transforming the landscape of CUP care.

## Conclusion

CUP are characterized by obscure primary origins and high heterogeneity, presenting significant challenges to traditional diagnostic and therapeutic approaches in TOO identification, personalized treatment, and prognostic assessment, which severely impact patient survival. Recent advances in AI technology provide effective new strategies for the clinical management of CUP. AI models based on molecular data enable high-precision TOO prediction through in-depth analysis of multi-omics features: DNA methylation models exhibit exceptional stability and specificity; targeted genomic sequencing models balance accuracy and clinical practicality; and circulating molecular data models pioneer non-invasive TOO tracing. AI models based on medical imaging complement the limited accessibility of molecular testing: pathological image AI models (e.g., TOAD, TORCH) demonstrate diagnostic performance comparable to clinical experts in CUP cohorts, while radiological AI models provide cost-effective solutions for TOO prediction in organ and bone metastases. Furthermore, AI applications in treatment response and prognosis prediction have initially achieved integrated decision support covering “diagnosis-treatment-prognosis.”

Despite challenges in data compatibility, model generalization, interpretability, and clinical translation, these bottlenecks are expected to be addressed through multi-modal data integration, interpretable AI innovation, and standardized database construction. In the future, AI will drive the transformation of CUP management from “experience-driven” to “precision and personalized,” serving as a core pillar of multidisciplinary collaborative care and providing robust support for improving patient outcomes.

## Data and code availability

Data and materials are available in the main paper and supplementary files.

## Acknowledgments

This work was financially supported by the 10.13039/501100016345Renmin Hospital of Wuhan University Cross-Innovation Talent Project (JCRCZN-2022–015).

## Author contributions

Conceptualization, Z.C.; resources, Z.C. and H.Y.; writing – original draft, Z.C., T.X., and H.Y.; review of the manuscript, H.Y., L.X., and J.Y.; funding acquisition, J.Y. All authors have read and approved the manuscript.

## Declaration of interests

The authors declare no competing interests.
